# Research on the Correlation between Breeding Environment and Activity of Yellow Feather Broilers Based on the Multichromatic Aberration Model

**DOI:** 10.1155/2021/2897879

**Published:** 2021-09-14

**Authors:** Zhonghao Zhao, Xiuguo Zou, Zhengling Yin, Zhibin Cao, Jie Zhang, Chenyang Wang, Wenchao Liu, Yungang Bai

**Affiliations:** ^1^College of Artificial Intelligence, Nanjing Agricultural University, Nanjing 210031, China; ^2^Jiangsu Province Engineering Laboratory of Modern Facility Agriculture Technology and Equipment, Nanjing 210031, China; ^3^College of Engineering, Nanjing Agricultural University, Nanjing 210031, China

## Abstract

Broiler behavior is closely related to the breeding environment. Therefore, studying broiler behavior helps breeding farm workers to better carry out welfare breeding. In the breeding environment of yellow feather broilers, temperature, humidity, and ammonia concentration are the main factors that affect the behavior of the broilers. This study used a multichromatic aberration model to process the color images of yellow feather broilers to extract the activity feature of the broilers at different periods, utilized the Cb component of YCbCr color model and the b component of Lab color model to remove background litter in images, and employed the Q component of YIQ color model to remove the feeders and the drinkers from the image. The segmented images were constructed into an accumulator to generate a heat map of yellow feather broilers' activity. Then, the correlation between the activity and the temperature and humidity index (THI) and the correlation between the activity and ammonia concentration were explored. The experiment found that the activity of the broilers was significantly positively correlated with ammonia concentration (*P* < 0.05), indicating that the activity of yellow feather broilers increased with ammonia concentration ascending. Besides, the THI in the broiler chamber had a significant positive correlation with the ammonia data (*P* < 0.01), indicating that when the THI in the broiler chamber increases, the ammonia concentration rises. The research provides a direction for exploring the impact of THI and ammonia concentration on the performance of yellow feather broilers. At the same time, it provides a theoretical basis for the early warning and judgment of broiler breeding by farm workers in the future.

## 1. Introduction

Animal behavior information is an important evaluation indicator of animal welfare. It can reflect the adaptation of the animal body to the environment help producers better manage livestock and poultry. Different broiler chamber environment conditions will also have different effects on the performance of broilers. Lower stocking density can improve the welfare index of broilers [1]. The extremely high level of stocking density will harm the performance of broilers, but other environmental factors are still important indicators that affect broiler production [2]. Abudabos et al. [3] found that high-density broiler breeding in a short time will significantly increase the stress response of broilers and damage the welfare of broilers. The improvement of environmental factors or the use of existing environmental resources can benefit the activity level of broilers, which will have an impact on the behavior of broilers [4]. Strengthening enrichment such as straw bales in the breeding environment can stimulate specific behavior of broilers, thereby enhancing animal welfare and generating economic benefits [5]. Among other environmental factors, light is considered to be a factor that directly affects broiler behavior. Since each broiler has a unique visual system and the stimulation of different light can have different effects on broiler behavior. Most studies have shown that blue light (450 nm) and green light (550 nm) have a positive effect on body weight of each broiler, while red light (700 nm) has a negative effect on broiler body weight and will stimulate the activity and aggressive behavior of broilers [6].

Heat stress is an important environmental factor that has many adverse effects on broiler breeding, including growth rate, weight, and meat quality. It also affects poultry welfare issues such as mortality [7]. Pereira et al. [8] proposed a temperature comfort clustering index, which can distinguish the differences in the aggregation of domestic under different temperature conditions. Li et al. [9] found that heat stress can significantly affect the daily behavior of broilers, including eating, drinking, and lying down. In addition, it will elongate the drinking and prone time of broilers. Ding et al. [10] combined transfer learning and target detection and used the constructed model to analyze the population characteristics of yellow feather broilers under heat stress. They found that after the temperature in the broiler chamber rises to 30℃, the activity index of the broilers will be reduced by 22.54% every three hours.

In the broiler chamber, ammonia is the main pollutant gas. The increase of air pollutants in the poultry houses will have a negative impact on the general production performance and own immune response of livestock and poultry [11]. Beker et al. [12] studied the effects of different ammonia concentrations on the production performance of broilers, tracheal diseases, and other physical and chemical properties. They discovered that the ammonia concentration in the broiler chamber would harass the performance of broilers and increase the susceptibility to diseases. Miles et al. [13] analyzed the impact of different levels of ammonia concentration on the performance of broilers and found that with the increase of ammonia concentration, the mortality of broilers rose. It can be seen that the ammonia concentration can affect the production performance of broilers. Therefore, to effectively control the ammonia concentration in the broiler chamber is necessary. Liu et al. [14] studied the values of ammonia at different concentrations and found that an increase in the value of ammonia concentration would raise the frequency of broilers to head shaking and damage the lung tissue of the broilers, which affects the performance of the broilers. Li et al. [15] found that low temperature and excessive ammonia would decrease egg production. In addition, the negative impact of ammonia on laying hens is more serious than low temperature.

During broiler production, sudden changes in behavior require manual early warning. The reality of broilers' rearing in groups, with the same appearance, high rearing density, and nonrigid body, makes it more challenging to study the behavior of broilers by means of manual detection than other large-sized animals. Since image processing technology has the advantages of noninvasive and real-time feedback, an increasing number of scholars have begun to use image processing technology to analyze and study broiler behavior. Various image processing methods have been successfully applied to broiler behavior analysis, and some substantive results have been achieved. Sergeant et al. [16] used image processing technology to segment the broiler images, determined the centroid coordinates of the broilers, and established the broiler's motion trajectory to achieve a better broilers' tracking effect. Dawkins et al. [17] used the optical flow algorithm to provide a more straightforward way to monitor the behavior of the flock, which can be applied to the monitoring of the entire life process of broilers. Aydin et al. [18] used digital image processing technology to normalize the image area occupied by each broiler. The Friedman test and Dunn test were used to conducting statistical analysis on the walking activities of the normal and abnormal broilers, and the relationship between the gait score and the degree of activity was obtained. Lao et al. [19] used machine vision algorithms to recognize the behavior of laying hens and track the activity and distribution of laying hens during the illumination period. Pereira et al. [20] chose the HSI color system to process the image and used erosion diffusion technology to extract the region of interest (ROI) in the image. Through image analysis, the area, circumference, and center of mass constituting the contour of the chicken could be acquired, which can be used to calculate displacement and velocity variables of the movement. Neves et al. [21] used image analysis and detection technology to obtain the effects of different feeding types on broiler behavior. Van Hertem et al. [22] developed a broiler gait score prediction model based on automatic measurement methods of flock distribution, activity level, and body weight. The experiment employs a camera system to continuously monitor the activity of broilers and obtain the activity level and distribution of the flock.

Since the breeding environment of the broiler chambers has a great influence on the growth of yellow feather broilers, it is necessary to study the correlation between the breeding environment and the behavior of yellow feather broilers. However, most studies combine ammonia concentration values with the physiological, physical, and chemical information of yellow feather broilers, but there are few more intuitive behavioral analysis studies.

In this paper, we take flat-raised yellow feather broilers as the research object. Firstly, based on the multichromatic aberration model, the collected yellow feather broiler images are segmented, and the image structure accumulator obtained by the segmentation is used to generate the yellow feather broilers' activity heat map. Then, we studied the impact of environmental factors such as ammonia, temperature, and humidity on the activity distribution of the yellow feather broilers.

## 2. Materials and Methods

### 2.1. Experimental Materials

**Figure 1:**
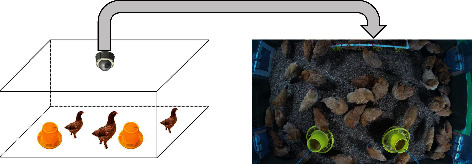
Real scene of the inside of the broiler chamber.

The experimental base is located in Jinniuhu Street, Nanjing, China. The coordinates of the experimental broiler chambers are longitude 118.52.6370 E, latitude 32.26.7686 N, and the standard is WGS84. Two experimental broiler chambers were placed symmetrically, which were recorded as Chamber 1 and Chamber 2, respectively. The length of broiler chamber is 1.9 m, and the width of broiler chamber is 2.9 m. The experimental data adopted the data extracted in Chamber 1.  Figure 1 shows a real scene of the inside of the broiler chamber. The video monitoring system used in the experiment included HIKVISION's CS-C4W-3C2WFR (2.8 mm) dome network camera, monitoring host, and network video recorder. The temperature and humidity detection system used in the experiment was the Renke temperature and humidity detection system, and the ammonia gas monitoring device, Renke ammonia transmitter, monitored the temperature and humidity data and the ammonia concentration value in the broiler chambers in real time to ensure the normal development of the experiment.

The experiment used a top-view camera device located directly above the center of each broiler chamber, 1.8 m from the ground. The image obtained by the camera can cover most of the space of the broiler chamber, which, however, cannot obtain part of the information near the 2.9 m long walls on both sides. The pixel size of the image collected by the camera was 3840 *∗* 2160. The experiment was carried out for a total of 38 days. We placed 19 broilers on December 5, 2019, and 26 broilers on December 10, 2019. When they were placed, the broilers were 75 days old and were in good physical condition. Placement in batches was to explore the effect of distribution density on the performance of yellow feather broilers. By the end of the experiment, there were 43 surviving broilers, and two yellow broilers died. LED light sources were used in the experimental broiler chambers, and the lighting time was 5:00–22:59 every day, and the lights in the broiler chambers were turned off during the rest of the day. The video capture device was utilized to intercept the image. The experiment selected three time periods of 6:00–6:59, 12:00–12:59, and 18:00–18:59 for collection to ensure that the activities of the yellow broilers in each time period are not affected by each other. At the same time, these three time periods can better represent the behavior of the three time periods in the morning, noon, and evening. One image was taken every minute, and a total of 180 images were collected in one day.

The environmental information at the air outlet of the inner circulation in the broiler chambers was the closest to that in the broiler chambers, so we deployed an ammonia collection device and a temperature and humidity acquisition device to collect the ammonia concentration and temperature and humidity data at the outlet of the inner circulation, respectively. In the broiler chambers, the initial ammonia concentration value was low, but the ammonia concentration value gradually rose as time accumulates. The data used in this article were the data collected from December 22, 2019 to January 3, 2020. In some periods, the ammonia data was missing due to human interference, so we selected the date when the data was complete for data analysis.

### 2.2. Color Model Analysis

Image segmentation is an important issue of image analysis and pattern recognition, which can determine the final analysis quality and recognition effect of the image [23]. During image processing, it is important to consider the performance of the method when images corrupted with noise and other imaging relics [24, 25]. As we all know, the images collected by the system are often displayed using the RGB color model, but due to the high correlation of the R, G, and B three components, that is, when the brightness changes, the three components will change accordingly, which is not suitable for image segmentation. The Lab color model is a uniform color space with a larger color gamut, which can reflect the difference in color values. The YCbCr color model is usually used for continuous image processing. The Cb and Cr components are the density offset components of blue and red, which are very useful on some occasions with uneven lighting. Because hue has nothing to do with highlights and shadows, it is very effective for distinguishing objects of different colors. The YIQ color model partially eliminates the correlation of RGB and is suitable for color image processing. Therefore, this study tried to use Lab, YCbCr, and YIQ color models to segment yellow feather broiler images.

**Figure 2:**
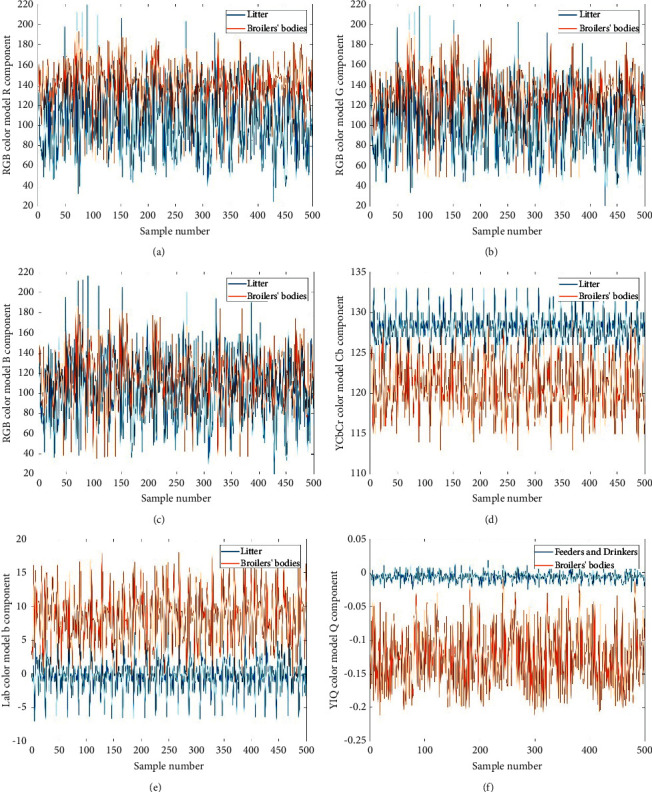
Component values of samples under different color models. (a) R component values of litter and broilers' bodies. (b) G component values of litter and broilers' bodies. (c) B component values of litter and broilers' bodies. (d) Cb component values of litter and broilers' bodies. (e) b component values of feeders and drinkers and broilers' bodies. (f) Q component values of feeders and drinkers and broilers' bodies.

Figures 2(a)–2(f) show the component values of the sample data set under different color models. Observing the collected images of the data set, it is found that the images are mainly composed of yellow feather broilers and complex backgrounds. The background mainly comprises black and white litter, yellow feather broilers' feeders, yellow feather broilers' drinkers, white water pipe and wall, and the broilers' bodies mainly composed of brown and white. In the image, the litter and the broilers' bodies occupy a larger area, and the color of the feeders and drinkers is similar to that of the yellow feather broilers. Therefore, the image data sets of litter, feeders, and broilers' bodies were extracted separately for segmentation. The experiment randomly selected ten images from the image data set collected from December 22, 2019, to January 3, 2020, and extracted 25 subimages for each type of litter, feeders, and broilers' bodies, and then extracted 100 pictures with a size of 20 *∗* 20 pixels for each type of picture as a sample data set. 500 pixels from the sample data set were randomly extracted for comparison, and different color models were utilized to compare and analyze the sample data sets. After comparing the data change curves of different components, we found that the Cb component of the YCbCr color model and the b component of the Lab model can better segment the broilers' bodies from the litter. And the Q component of the YIQ color model can better separate the broilers' bodies and the feeders. Figures 2(a)–2(c) show the R, G, and B component values of the litter and broilers' bodies, respectively.  Figure 2(d) shows the Cb component values of litter and broilers' bodies. Figures 2(e) and 2(f) show the b component values of feeders and drinkers and broilers' bodies, respectively. It is observed clearly that the attribute values in the left column are all highly coincident, but the attribute values in the right column have an obvious boundary, respectively.

### 2.3. Image Segmentation Based on the Multichromatic Aberration Model

Based on the above analysis of different color models of yellow feather broiler images, the experiment used the Cb component and b component thresholds to remove the litter background and then used the Q component threshold to segment the feeders and the broilers' bodies. The calculation of the threshold can be regarded as a two-classification problem. In the extracted sample data points, an appropriate threshold can well separate the component values of different data samples. Different threshold selection methods will produce different processing effects. When selecting the threshold, the idea based on maximizing the segmentation interval will be affected by the distribution range of the single-class samples, while the idea based on clustering is to ensure that objects within a class after classification maximize their homogeneity, and for heterogeneous objects, maximize their heterogeneity. In this paper, our idea was based on clustering. When two types of data points are known, we selected different thresholds to ensure that the number of correctly classified samples was the largest. The calculation equations for the threshold are as follows:

(1) $$\begin{array}{c} T_{b}^{*}=\;\underset{T_{b}}{\arg\max}\left(\overset{100}{\underset{i=1}{\sum }}\left(I_{b1i}<T_{b}\right)+\overset{100}{\underset{i=1}{\sum }}\left(I_{b2i}>T_{b}\right)\right),\\ T_{Cb}^{*}=\;\underset{T_{Cb}}{\arg\max}\left(\overset{100}{\underset{i=1}{\sum }}\left(I_{Cb1i}>T_{Cb}\right)+\overset{100}{\underset{i=1}{\sum }}\left(I_{Cb2i}<T_{Cb}\right)\right),\\ T_{Q}^{*}=\;\underset{T_{Q}}{\arg\max}\left(\overset{100}{\underset{i=1}{\sum }}\left(I_{Q2i}<T_{Q}\right)+\overset{100}{\underset{i=1}{\sum }}\left(I_{Q3i}>T_{Q}\right)\right), \end{array}$$

where *T*_b_, *T*_Cb_, and *T*_Q_ represent the thresholds of the b component, Cb component, and Q component, respectively; I_b1*i*_ represent the values of the b component of the Lab model that randomly extracts the *i*th sample data point of the litter, I_b2*i*_ represent the values of the b component of the Lab model that randomly extracts the *i*th sample data point of the broilers' bodies, I_Cb1*i*_ represent the values of the b component of the YCbCr model that randomly extracts the *i*th sample data point of the litter, I_Cb2*i*_ represent the values of the b component of the YCbCr model that randomly extracts the *i*th sample data point of the broilers' bodies, I_Q2*i*_ represent the values of the b component of the YIQ model that randomly extracts the *i*th sample data point of the broilers' bodies, and I_Q3*i*_ represent the values of the b component of the YIQ model that randomly extracts the *i*th sample data point of the feeders.

(2) $$\begin{array}{c} H=\left\{\begin{array}{cc} 1, & \text{if }x_{\text{Cb}}>T_{\text{Cb}}\text{ and }x_{b}<T_{b}\text{ and }x_{Q}<T_{Q},\\ 0, & \text{otherwise,} \end{array}\right. \end{array}$$

where *H* represents the image area divided by the Cb component, the b component, and the Q component and *x* is the pixel in the image.

**Figure 3:**
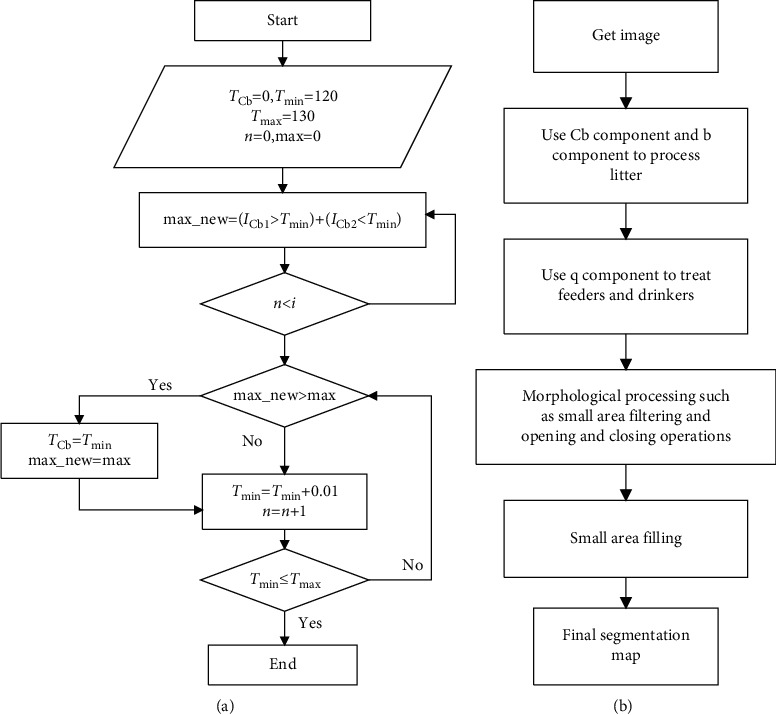
Technical flowchart of image segmentation. (a) Color model threshold calculation flowchart. (b) Image segmentation flowchart.

In the first segmentation process, using the YCbCr and Lab color models can remove the most of the background image, including litter and wall information. Using the YIQ color model, the Q components of the broilers' bodies and the feeders no longer overlap. The Q component is used to divide the broilers' bodies and the feeders. Finally, using morphological processing such as small area filtering and opening and closing operations, the yellow feather broilers can be better segmented.  Figure 3(a) shows the Cb component threshold calculation flowchart, *i* is the number of sample data points, I_Cb1_ is the Cb component value of the litter, and I_Cb2_ is the Cb component value of the broilers' bodies. By updating the threshold, we can get the most appropriate parameter setting, which makes the sum of the two attribute values on both sides of the boundary be the most. Every color component segmentation threshold used this method.  Figure 3(b) shows the flowchart of image segmentation.

### 2.4. Correlation Analysis

The correlation coefficient can reflect the strength of the statistical relationship between two random variables. It is widely used in various scientific and technical research fields. And it is of great significance for analyzing the correlation between different impact factors. Xu [26] summarized the research on correlation coefficients. In order to characterize different correlations, there are three classic processing methods, namely, Pearson's Product Moment Correlation Coefficient (PPMCC) proposed by the founder of statistics K. Pearson, Spearman's rank correlation coefficient (Spearman's rho, SR) proposed by the psychologist Spearman, and Kendall's rank correlation coefficient (Kendall's tau, KT) proposed by the statistician Kendall. In this paper, Kendall's rank correlation coefficient is used to analyze the temperature and humidity index, ammonia concentration, and activity. The Kendall correlation coefficient is defined as follows: let *X*_*i*_, *Y*_*i*_, *i* = 1, 2,…, *n*, represent *n* pairs of mutually independent data. The data points obey a certain binary continuous distribution. Arrange the sequence *X*_*i*_ in ascending order to obtain a new set of data pair sequence (*X*_*i*_, *Y*_*i*_), where *X*_1_ to *X*_*n*_ are the order statistics about *X*; corresponding *Y*_*i*_ is called the companion of *X*_*i*_. Let sgn(·) be a symbolic function. The Kendall correlation coefficient KT (*r*_*k*_) is defined as follows:

(3) $$\begin{array}{c} \begin{array}{c} r_{k}\left(X_{i},Y_{i}\right)≜\frac{\sum_{i=1}^{n}\sum_{j=1}^{n}\mathrm{sgn}\left(X_{i}-X_{j}\right)\mathrm{sgn}\left(Y_{i}-Y_{j}\right)}{n\left(n-1\right)} \end{array}. \end{array}$$

In this paper, the data collected in the three time periods on different dates were, respectively, summarized and analyzed. For the ammonia concentration data, the average value of the ammonia concentration in one hour represented the ammonia concentration in the time period, and the temperature and humidity data adopted the same processing method. After processing the collected images, the experiment accumulated the number of times that the segmented yellow feather broiler images appeared at different positions. 0 to 60 represented the number of times the yellow feather broilers appeared at this position. The experiment selected the ratio of pixels with 10–49 occurrences to the total pixels of a single image as the activity information of the yellow feather broilers in this time period. We used Kendall's rank correlation coefficient to analyze the correlation of the above data information.

## 3. Results and Discussion

### 3.1. Image Segmentation Result

The computer used for image processing in this paper is configured as Intel^®^ Core™ i5-CPU central processing unit. The operating system uses Windows 10 (64-bit), 4GB memory, and MATLAB R2018a platform for image preprocessing and extraction sample data set.

**Figure 4:**
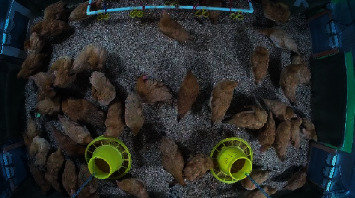
Image processing original image.

**Figure 5:**
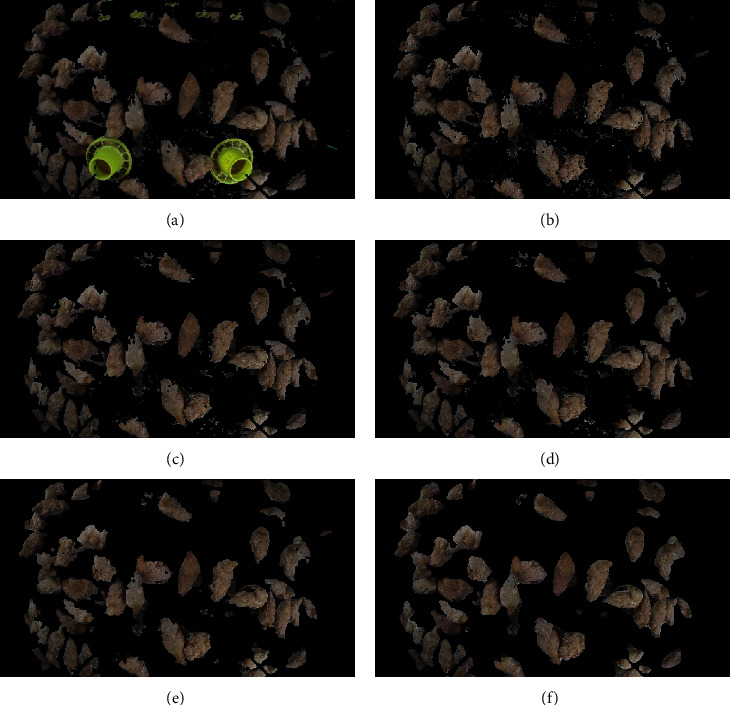
Segmentation method process diagram. (a) Litter treatment result. (b) Feeders and drinkers process result. (c) Small area filtering result. (d) Closed operation result. (e) Open operation result. (f) Small area filling result.

Based on this paper's method, the 60 images after segmentation were accumulated in each time period. Then, we can obtain the distribution heat map of yellow feather broilers in this period. This paper used direct observation to judge the final segmentation effect.  Figure 4 shows the original image of the processed image. Figures 5(a)–5(f) show the effect of the image segmentation process.

Figure 5(a) shows that the Cb component and *b* component are used to segment the collected image, which can eliminate the background material and most of the wall information.  Figure 5(b) shows that the *Q* component of the YIQ model can be used to eliminate feeders and drinkers from the figure. Figures 5(c)–5(e) show that morphological processing such as small area denoising and opening and closing operations on the image with the background information removed can get most of the yellow feather broilers' bodies image. The processed image is filled in a small area again, and the small area of the cavity on the yellow feather broilers' bodies image is filled. The final processing effect is shown in Figure 5(f).

### 3.2. Results of Aquaculture Environment Data Collection

Regarding the collected ammonia data, the average value of the collected ammonia concentration data in the required time period was taken as the ammonia concentration value in this time period.  Table 1 shows the changes in the obtained ammonia concentration values in different time periods.

According to the chart, it can be found that the ammonia concentration is generally higher at 6:00–6:59 in the morning. This is because the experimenters cleaned the yellow feather broilers' manure and litter during the day, and no one interfered in the broiler chamber at night.

At the same time, the experiment used the temperature and humidity sensor installed at the air outlet of the inner circulation to collect the air temperature (*T*) and relative humidity (RH) of the time period corresponding to the change of the ammonia concentration value. Similarly, we took the average of the temperature and humidity data in the required time period as the temperature and humidity data in this time period. Then, we calculated the temperature and humidity index in the chamber in different time periods. The temperature and humidity index (THI) calculation formula is shown in equation (4) [27], and calculation results are shown in Table 2.

(4) $$\begin{array}{c} \begin{array}{c} \text{THI}=\left(1.8\times T+32\right)-\left(0.55-0.0055\times \text{RH}\right)\times \left(1.8\times T-26\right) \end{array}, \end{array}$$

where *T* is the air temperature (°C) and RH is the relative humidity (%).

### 3.3. Correlation Analysis

**Figure 6:**
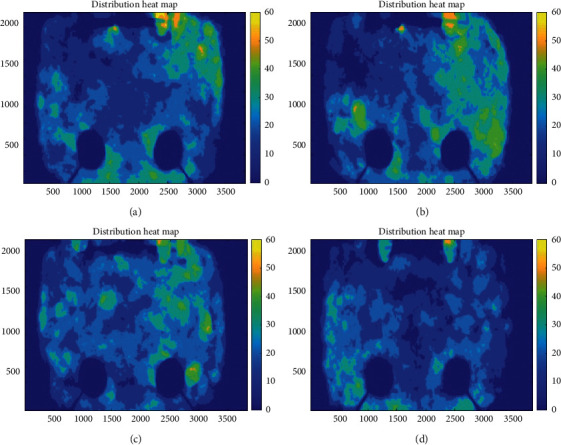
Distribution map of yellow feather broilers in different time periods. (a) 2019.12.22 12:00–12:59, THI: 58.48, ammonia: 29.4 ppm. (b) 2019.12.22 18:00–18:59, THI: 58.10, ammonia: 23.55 ppm. (c) 2019.12.23 12:00–12:59, THI: 57.33, ammonia: 26.98 ppm. (d) 2019.12.29 18:00–18:59, THI: 57.88, ammonia: 32.15 ppm.

The experiment selected data from December 22, 2019 to January 3, 2020 for analysis. First of all, based on the environmental impact of different ammonia and temperature and humidity, we used subjective analysis to observe the distribution heat maps of yellow feather broilers on different days at the same time period and at different time periods on the same date. Then, we accumulated the number of times that the segmented yellow feather broiler images appear in different positions to obtain the distribution heat map of the yellow feather broilers in different time periods. Through constructing into an accumulator, we can get a heat map of yellow feather broilers' activity.  Figure 6 shows the distribution of yellow feather broilers' activity in different time periods. 0 to 60, respectively, represent the number of times the yellow feather broilers appears at this position. It can be seen from the result graph that when the THI difference is small in the same time period, the activity of the yellow feather broilers increases with the ammonia concentration, and the aggregation decreases.

In the experiment, the correlation between THI of the broiler chamber and the activity of the yellow feather broilers and the correlation between the ammonia concentration of the broiler chamber and the activity were explored. We processed the collected data and then used the Kendall correlation coefficient to explore the interrelationship between them.  Table 3 shows the correlation between the THI and the ammonia concentration in the broiler chamber.

According to the Kendall correlation coefficient analysis, the THI and the ammonia concentration have a significant correlation (*P* < 0.01). At the same time, based on the data of this experiment, it can be found that the THI and the ammonia concentration are numerically related. This feature of the two also provides ideas for controlling the ammonia concentration in the broiler chamber in the future. Placing ventilators in different positions can control the environment in the broiler chamber more effectively.

In the experiment, one picture was taken every minute for a period of time, a total of 60 pictures per hour. Taking into account the improper image processing and the situation of yellow feather broilers gathering and lying for a long time, the experiment took the ratio of the image area occupied by the number of distributions in the heat map between 10 and 49 to the total area of the image as the distribution index of the yellow feather broilers.  Table 4 shows the correlations between THI, ammonia concentration, and the yellow feather broilers' activity.

From the results of correlation between THI, ammonia concentration, and activity, we get the following conclusions:

1. There is a significant correlation between THI and the activity of yellow feather broilers (*P* < 0.05). That is, as the THI increases, the activity of yellow feather broilers increases.
2. There is a significant correlation between the ammonia concentration and the activity of yellow feather broilers (*P* < 0.05). That is, as the ammonia concentration increases, the activity of yellow feather broilers increasing.

It can be seen that the increase in ammonia concentration has an impact on the production performance of yellow feather broilers. Moreover, higher activity may lead to lower feed conversion rates of yellow feather broilers and reduce broilers' quality. This paper provides a new analysis direction for future broiler breeding researchers.

## 4. Conclusions

The broiler breeding environment has an important impact on broiler welfare. The behavior of broilers can reflect its adaptability to the environment. In the broiler chamber, many environmental factors affect yellow feather broilers, including temperature and humidity, ammonia concentration, light frequency, and stocking density. It is of great significance to study and analyze the impact of environmental factors on yellow feather broilers.

Noninvasive research methods such as image processing can be used to analyze without affecting the lives of broilers. In the analysis of broilers, using image technology, machine learning, and other technologies can help farmers analyze the welfare of broilers better.

As the main indicators in the breeding environment, THI and ammonia concentration have a significant impact on the behavior of yellow feather broilers. Aiming at the influence of THI and ammonia concentration on the behavior of yellow feather broilers, this paper proposes a yellow feather broilers' activity analysis method based on a multichromatic aberration model. Using the multichromatic aberration model to process the yellow feather broiler images can effectively segment the yellow feather broilers from the image. According to image segmentation results, we can obtain heat maps of the activity distribution of yellow feather broilers in different periods. It is found that the activity of the yellow feather broilers increases with the THI and the ammonia concentration. The performance result shows that the overall resting degree of the yellow feather broilers in the broiler chamber decreases within a certain period of time, and the range of activities is wider. At the same time, based on the collected environmental data, it is found that there is a significant correlation between the THI and ammonia concentration data in the broiler chamber. In the future, researchers should use the activity distribution index of broilers as an analysis indicator, which can help analyze the impact of environmental factors on broiler production performance. Therefore, the breeder should monitor and control the temperature, humidity, and ammonia concentration of the broiler chamber at a suitable limit. When the activity of yellow feather broilers in the broiler chamber is abnormal, the breeder should make timely early warning judgments to prevent economic production losses due to excessive ammonia gas concentration.

## Figures and Tables

**Table 1:** Ammonia concentration in each time period (unit: ppm (parts per million)).

Date period	22.12.2019	23.12.2019	24.12.2019	25.12.2019	26.12.2019	29.12.2019	2.1.2020	3.1.2020
6:00–6:59	50.00	25.23	33.47	44.92	38.44	24.36	19.88	23.34
12:00–12:59	29.40	26.98	21.75	35.63	30.46	22.55	15.60	09.14
18:00–18:59	23.55	31.26	21.93	44.07	46.62	32.15	26.81	26.55

**Table 2:** THI in each time period.

Date period	22.12.2019	23.12.2019	24.12.2019	25.12.2019	26.12.2019	29.12.2019	2.1.2020	3.1.2020
6:00–6:59	61.45	52.59	56.34	60.97	57.33	55.48	53.13	55.95
12:00–12:59	58.48	57.33	55.04	58.91	54.99	59.32	54.48	51.25
18:00–18:59	58.10	57.03	54.98	61.02	55.97	57.88	56.43	56.84

**Table 3:** Correlation between broiler chamber THI and ammonia concentration.

	Attributes	NH_3_
THI	Correlation coefficient	0.507^*∗∗*^
	Statistical significance	0.001
	Number of cases	24

^*∗∗*^*P* < 0.01, significant correlation.

**Table 4:** Correlations between THI, ammonia concentration, and activity.

	Attributes	THI	NH_3_
Activity	Correlation coefficient	0.375^*∗*^	0.307^*∗*^
	Statistical significance	0.013	0.043
	Number of cases	24	24

^*∗*^*P* < 0.05, significant correlation.

## Data Availability

A link to online repositories and deposition codes can be provided where applicable.
